# Sensitivity to increase in leaf endogenous ABA is responsible for stomatal closure under drought stress in cowpea (***Vigna unguiculata*** (L.) Walp.)

**DOI:** 10.1080/15592324.2025.2598081

**Published:** 2025-12-08

**Authors:** Nao Murakami, Ryuki Tamaki, Yuji Nakamura, Shino Mikuriya, Jihun Kim, Chetphilin Suriyasak, Yushi Ishibashi

**Affiliations:** a Graduate School of Bioresource and Bioenvironmental Sciences, Kyushu University, Fukuoka, Japan; b Faculty of Agriculture, Kyushu University, Fukuoka, Japan

**Keywords:** Drought stress, abscisic acid, stomatal closure, sensitivity, soybean

## Abstract

Drought stress is a major environmental factor limiting crop productivity worldwide. Plants respond to drought through various physiological mechanisms, including stomatal closure mediated by abscisic acid (ABA). This study investigated the relationship between leaf ABA content and stomatal closure in drought-tolerant cowpea (*Vigna unguiculata*) and drought-sensitive soybean (*Glycine max*). Under drought conditions, stomatal conductance decreased faster in cowpea than in soybean, significantly by day 2. Leaf ABA content increased earlier in cowpea, suggesting a strong correlation between ABA accumulation and stomatal closure. In contrast, both stomatal conductance and ABA accumulation were delayed in soybean. A lower ABA concentration was required to induce stomatal closure than in soybean, indicating that stomatal sensitivity to ABA was higher in cowpea. These findings suggest that cowpea’s superior drought tolerance is due to its rapid and more sensitive ABA-mediated stomatal response and provide insights for improving drought resilience in soybean through targeted breeding or biotechnological approaches.

## Introduction

Environmental stresses have multiple effects on plant growth. Drought is the most serious of the environmental stresses that limit global crop production; for example, drought reduces yields of soybean (*Glycine max* (L.) Merr.) by 46%–71%.^1^^,^^2^ The cost of drought damage around the world is estimated at USD 307 billion per year.^3^ In order to thrive under drought stress, plants use sophisticated responses and adaptations, including stomatal closure to prevent water loss, accumulation of osmolytes, and ROS scavenging.^2^^,^^4^^,^^5^

Abscisic acid (ABA) is a key phytohormone that mediates drought stress responses in plants, and drought stress induces the expression of ABA biosynthesis genes, leading to ABA accumulation.^6^^,^^7^ Our previous study shows that rapid ABA biosynthesis in roots is associated with stomatal closure under drought stress in cowpea.^8^ Although ABA from roots is important,^9-12^ previous studies have shown that ABA synthesized in leaves is essential for stomatal closure as activation of ABA-inducible promoters in leaves of *Arabidopsis thaliana* (L.) requires reduction of water potential^13^^,^^14^ ABA triggers a signaling cascade in the guard cells, resulting in stomatal closure and reduction of water loss via transpiration.^15^ Leaf stress of PEG treatment significantly leads to an immediate increase in leaf ABA level, leading to rapid stomatal closure in peanut (*Arachis hypogaea* L.) leaves.^16^ These reports indicate that water stress is first recognized via a change in the water status in leaves, regardless of whether the roots are exposed to water stress or not, and then water stress activates *de novo* ABA biosynthesis in leaves to close the stomata. Therefore, the effects of ABA in leaves on stomatal closure under drought stress are considered to be crucial for stomatal closure.^13^^,^^14^^,^^17-19^

Cowpea (*Vigna unguiculata* (L.) Walp.) is a legume crop of African origin, grown for food and forage, with a production of 9.8 million t worldwide, 96% of it in Africa, where rainfall is unreliable.^20^ Cowpea survives better under drought stress than other crops, for example, soybean, maize, sorghum, pearl millet, tomato, and sunflower.^21^^,^^22^ One of the mechanisms of its drought tolerance is rapid stomatal closure under drought stress.^21^^,^^23^ Cowpea’s higher root ABA content accumulated under drought stress is negatively correlated with leaf stomatal conductance, evidence that root ABA content plays an important role in stomatal closure.^8^^,^^24^ However, whether leaf ABA content regulates stomatal closure and their correlation under drought stress remains to be elucidated.

Here, we investigated the correlation between stomatal closure and leaf ABA content in drought-tolerant cowpea and drought-sensitive soybean under drought to identify which mechanisms of cowpea can be introduced into soybean.

## Materials and methods

### 1. Plant materials and growth condition

Seeds of cowpea line ‘IT98K-205-8’ and soybean ‘Fukuyutaka’ were sown in nursery pots (ø 130 mm × H 113 mm) filled with sand containing 1.25 g of compound fertilizer (N:P:K = 3:10:10) and 1.25 g magnesium lime in the glasshouse at Kyushu University, Japan (33.5976° *N*, 130.2242° E), on 28 June 2024. After the second trifoliate leaves were fully expanded, we applied drought stress by stopping watering for 4 days; during this period, the soil was covered with aluminum foil to limit soil water evaporation. The first trifoliate leaves were sampled on days 0 to 4 after treatment (DAT) at 10:00.

### 2. Measurement of pot weights, soil water content and leaf relative water content

Pot weights were measured using a mechanical upper-dish scale (Daiwa Scale Co.) between 8:00 and 9:00. About 3 g of soil was taken from the top and center of each pot and fresh weight was measured, after drying at 80 °C for at least 96 hours, the dry weight was measured. The soil moisture content at the top and center of the pot was determined, and the average value was taken as the soil moisture content of the individual.

Soil water content(%)=(fresh weight–dry weight)×100/fresh weight

After measuring the fresh weight (FW) of the first leaf, the chopped samples were soaked in a zipper bag with distilled water and incubated at 4 °C for 24 hours. The turgid weight (TW) was measured, and the samples were dried at 80 °C for at least 72 hours for dry weight (DW).

Relative water content(%)=(FW–DW)×100/(TW–DW)

### 3. Stomatal conductance measurement

A portable leaf porometer (SC-1, Shinko Seiki Co., Ltd.) was used to measure the stomatal conductance in the second trifoliate leaves between 08:30 and 10:00. Relative stomatal conductance was calculated as observed stomatal conductance divided by the maximum stomatal conductance observed for each species (one plant as a biological replicate).

### 4. Leaf area and stomatal density measurements

Leaf area was measured using ImageJ and stomatal density was measured using epidermis imprints as described previously.^25^ Stomata were observed under BZ-X170 (Keyence) and counted using BZ-X Analyzer v. 1.3.1.1 software.

### 5. Quantification of endogenous ABA

To quantify leaf endogenous ABA, we analyzed leaf tissue (50–100 mg DW) by LC-MS/MS as described.^26^ D_6_-ABA purchased from OlChemIm (Olomouc) was used as an internal standard. Leaf samples were freeze-dried for 24 h, powdered, and extracted with 90% MeOH overnight at 4 °C. ABA was analyzed on a quadrupole time-of-flight mass spectrometer (QTOF X500B, AB Sciex) equipped with an electrospray ionization source coupled with an ultrahigh-performance liquid chromatography instrument (ExionLC AD, Sciex). An Acquity UPLC BEH C18 column (1300 Å, 1.7 μm, 2.1 mm × 100 mm), Oasis WAX cartridges (3 CC, 100 mg), and Oasis MCX cartridges (3 CC, 100 mg) were purchased from Waters (Milford, MA, USA). Data are presented as means of five or six replications (one plant as a biological replicate).

### 6. RNA extraction and quantitative real-time PCR

Total RNA was isolated from leaves of soybean and cowpea using the SDS–phenol–LiCl method.^27^ cDNA was synthesized from the RNA template with ReverTra Ace reverse transcriptase (Toyobo, Osaka, Japan) according to the manufacturer’s instructions. Quantitative real-time PCR was performed in a CFX Connect Optics Module real-time PCR detection system (Bio-Rad, Hercules, CA, USA) with Thunderbird SYBR qPCR mix (Toyobo) according to the manufacturer’s instructions. Thermal cycling conditions were as follows: initial denaturation at 94 °C for 2 min, followed by 40 cycles of denaturation at 94 °C for 20 s, annealing at a primer-specific temperature for 20 s, and extension at 72 °C for 20 s. The *GmEF1b* and *VuEF1b* gene was used for normalization in soybean and cowpea, respectively. Primer sequences are listed in Table S2 supplemental file.

### Data and statistical analysis

Statistical analyzes were performed in SPSS v. 28.0.0.0 software (IBM). Differences among treatments were analyzed by two-tailed Student’s *t*-test and Tukey’s test. The regression analysis between stomatal conductance and leaf endogenous ABA content was performed using raw data after log transformation of relative stomatal conductance. The regression curves were plotted as described^28^ to the data using SPSS v. 28.0.0.0 software (IBM). The goodness of fit was evaluated using the coefficient of determination (r^2^) and residual analysis.

## Results and discussion

**Figure 1.**
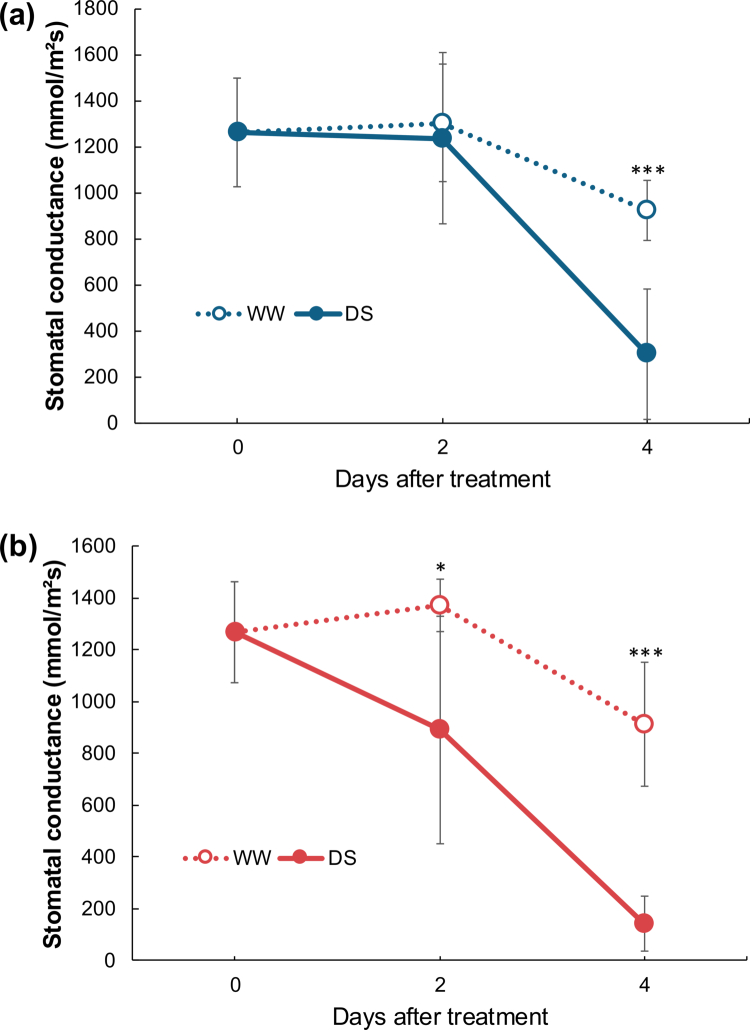
Stomatal conductance in leaves of (a) soybean and (b) cowpea exposed to 0, 2, or 4 days of either well watering (WW) or drought stress (DS). Error bars, SD (5 or 6 biological replicates). *p < 0.05, ***p < 0.001 (Student’s *t*-test).

Under drought stress, soybean stomata were still fully open at 2 DAT (Figure 1a). In contrast, cowpea stomata had already started to close at 2 DAT, and conductance was significantly lower than that of well-watered leaves (Figure 1b). These results show the much faster drought-induced stomatal closure of cowpea than that of soybean, as reported previously,^23^ although pod weight and soil water contents under drought stress did not differ between soybean and cowpea (Figure S1 supplemental file).

**Figure 2.**
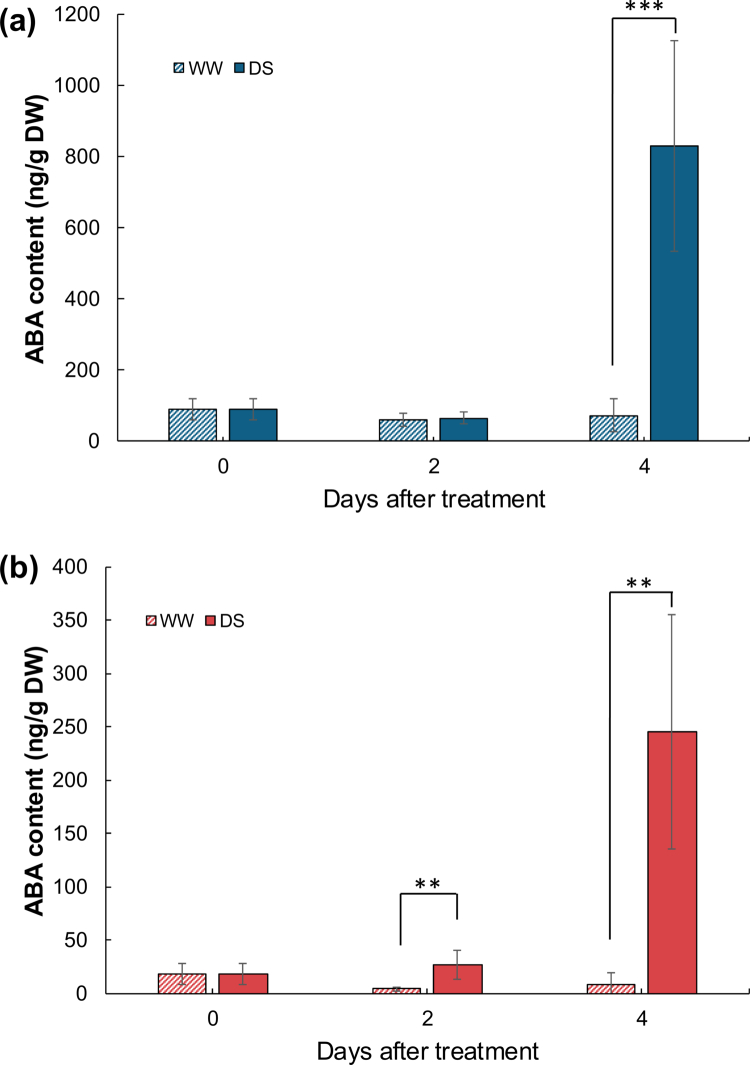
ABA content in leaves of (a) soybean and (b) cowpea exposed to 0, 2, or 4 days of well watering (WW) or drought stress (DS). Error bars, SD (5 or 6 biological replicates). **p < 0.01 (Student’s *t*-test).

ABA content in drought-stressed leaves of both species increased significantly by 4 DAT, to 9 × that in well-watered leaves in soybean and 13 × that in cowpea (Figure 2). In drought-stressed soybean leaves, ABA content had not changed by 2 DAT, but it was significantly greater than in well-watered leaves at 4 DAT (Figure 2a). In drought-stressed cowpea leaves ABA content was already significantly greater than in well-watered leaves by 2 DAT, when stomatal closure occurred (Figure 2b). These results suggest that a rapid increase in ABA content in cowpea leaves induces the rapid stomatal closure under drought stress. ABA is synthesized not only in Arabidopsis roots and transported to the shoots, but also in leaves under drought,^29^ suggesting the importance of leave-sourced ABA in cowpea under drought stress for stomatal closure.

**Figure 3.**
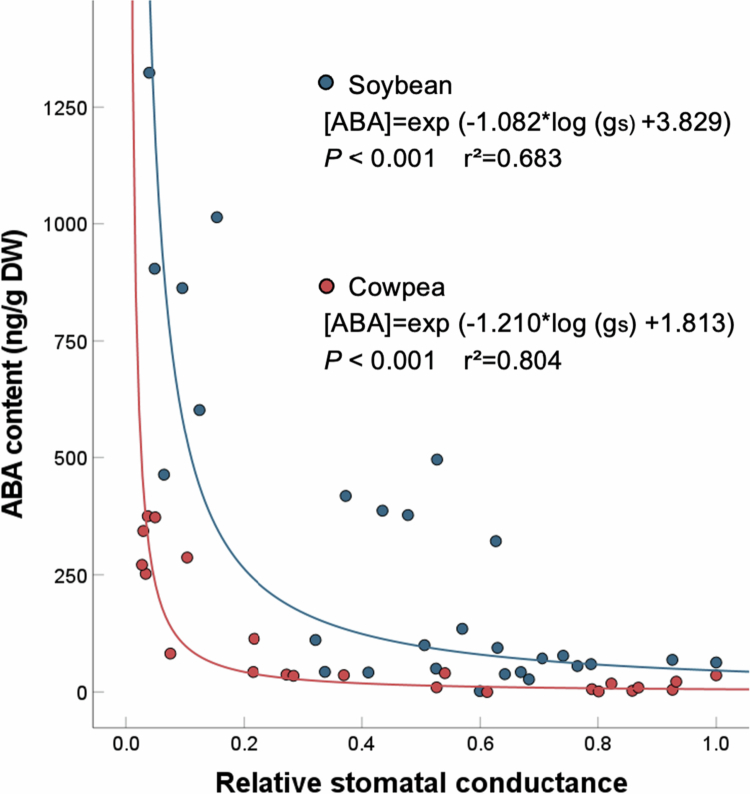
Correlations between relative stomatal conductance and ABA content in leaves of soybean (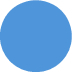) and cowpea (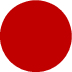) exposed to 0–4 days of drought stress. The dotted curves are approximations.

Although ABA increased rapidly in cowpea leaves by 2 DAT, its absolute amount was low—about one-third that in soybean. This difference suggests that cowpea can close its stomata even at a low ABA content, and that it is more sensitive to ABA than soybean. The strong association of the decrease in stomatal conductance with an increase in leaf ABA content in both species (Figure 3) suggests that the accumulation of ABA in the leaf induced stomatal closure. Soybean accumulated ~100 ng/g DW of ABA at 50% stomatal closure, whereas cowpea needed just 15 ng/g DW to do the same. Since different species have different leaf morphology and stomatal densities, ABA contents under drought stress were expressed per leaf and per stomata, indicating that compared to soybean, cowpea showed only about 25% and 20% of ABA contents per leaf and per stomata, respectively (Table S1 supplemental file). These results suggested high sensitivity to lower ABA level for stomatal closure in cowpea. In addition, relative expression of genes involved in ABA signaling in soybean and cowpea leaves under drought was analyzed. The results showed that ABA signaling gene expression in soybean was not yet induced at 2 DAT, however, *VuSnRK2.6* (SNF-related protein kinase2) was induced by 1.25-fold in cowpea leaves (Figure S2 supplemental file). SnRK2 protein is conserved in many plant species, which is activated by ABA for stomatal closure.^30^^,^^31^ Consequently, due to rapid and higher sensitivity of ABA for stomatal closure, cowpea leaves maintained higher relative water content compared to soybean under drought stress (Figure S3 supplemental file). Another study also suggest the role of ABA sensitivity for drought tolerance, as the Mg-chelatase H subunit (CHLH) mediates chlorophyll biosynthesis and mediates ABA-induced stomatal closure: overexpression of CHLH in guard cells enhanced the sensitivity of Arabidopsis stomatal guard cells to ABA, increasing drought tolerance.^32^

In all, we suggest that high sensitivity to ABA is one of the drought tolerance mechanisms of cowpea, as we also observed that cowpea leaves maintained higher relative water content Although stomatal closure in cowpea is correlated with root ABA levels under drought,^8^^,^^24^ our results in this study shows that stomatal closure is also positively correlated with leaf ABA content. It is necessary to further elucidate the mechanism of ABA transport from roots to leaves as well as ABA responses in leaves.

## Conclusion

Cowpea stomata closed rapidly under drought stress, limiting water loss through transpiration (Figure 1), and the leaf ABA content increased at the same time (Figure 2). The rapid stomatal closure appears to be due to the increased leaf ABA content, which was lower than in soybean leaves. The higher sensitivity of cowpea to ABA than that of soybean (Figure 3) explains its drought tolerance.

## Supplementary Material

Supplementary Material SF revise first final
