# Supplementary material for: Sensitivity to increase in leaf endogenous ABA is responsible for stomatal closure under drought stress in cowpea (Vigna unguiculata (L.) Walp.)
Source: Plant Signal Behav. 2025 Dec 8;20(1):2598081. doi: 10.1080/15592324.2025.2598081 (PMC12688217; doi:10.1080/15592324.2025.2598081)
Supplement: Supplementary Material — SF revise first final [file KPSB_A_2598081_SM5814.docx]

Figure S1. Pot weight (a) and soil water content (b) of soybean and cowpea plant samples under well-watered (WW) and drought stress (DS) conditions. Error bars represent SD values (five or six biological replicates).

**(a)**

**(b)**

Figure S2. Relative gene expression of ABA signaling-related genes in leaves of (a) soybean and (b) cowpea at 2 DAT under well-watered (WW) or drought stress (DS) conditions. Gene expression of DS was normalized to that of WW showing fold-change of each gene. Error bars represent SD values (five to six biological replicates).

**(b)**

**(a)**

Figure S3. Relative leaf water contents of (a) soybean and (b) cowpea at 4 DAT under well-watered (WW) or drought stress (DS) conditions. Error bars represent SD values (five or six biological replicates), *P* < 0.05* according to student’s *t*-test.


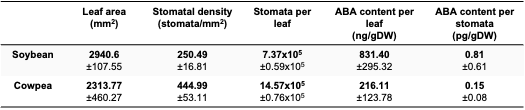


Values represent the mean ± SD (four to seven biological replicates).

Table S1. Average leaf area, stomatal density, average stomata per leaf, ABA content per leaf and ABA content per stomata of soybean and cowpea exposed to drought stress at 4 DAT.


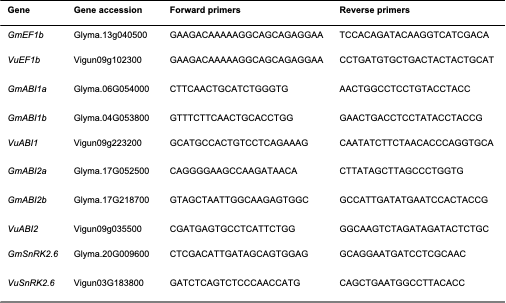


Table S2. qRT-PCR primers used in this study
